# DNA Repair Repertoire of the Enigmatic Hydra

**DOI:** 10.3389/fgene.2021.670695

**Published:** 2021-04-29

**Authors:** Apurva Barve, Alisha A. Galande, Saroj S. Ghaskadbi, Surendra Ghaskadbi

**Affiliations:** ^1^Developmental Biology Group, MACS-Agharkar Research Institute, Pune, India; ^2^Centre of Excellence in Science and Mathematics Education, Indian Institute of Science Education and Research (IISER), Pune, India; ^3^Department of Zoology, Savitribai Phule Pune University, Pune, India

**Keywords:** hydra, DNA repair, evolution, xeroderma pigmentosum, nucleotide excision repair pathway, base excision repair

## Abstract

Since its discovery by Abraham Trembley in 1744, hydra has been a popular research organism. Features like spectacular regeneration capacity, peculiar tissue dynamics, continuous pattern formation, unique evolutionary position, and an apparent lack of organismal senescence make hydra an intriguing animal to study. While a large body of work has taken place, particularly in the domain of evolutionary developmental biology of hydra, in recent years, the focus has shifted to molecular mechanisms underlying various phenomena. DNA repair is a fundamental cellular process that helps to maintain integrity of the genome through multiple repair pathways found across taxa, from archaea to higher animals. DNA repair capacity and senescence are known to be closely associated, with mutations in several repair pathways leading to premature ageing phenotypes. Analysis of DNA repair in an animal like hydra could offer clues into several aspects including hydra’s purported lack of organismal ageing, evolution of DNA repair systems in metazoa, and alternative functions of repair proteins. We review here the different DNA repair mechanisms known so far in hydra. Hydra genes from various DNA repair pathways show very high similarity with their vertebrate orthologues, indicating conservation at the level of sequence, structure, and function. Notably, most hydra repair genes are more similar to deuterostome counterparts than to common model invertebrates, hinting at ancient evolutionary origins of repair pathways and further highlighting the relevance of organisms like hydra as model systems. It appears that hydra has the full repertoire of DNA repair pathways, which are employed in stress as well as normal physiological conditions and may have a link with its observed lack of senescence. The close correspondence of hydra repair genes with higher vertebrates further demonstrates the need for deeper studies of various repair components, their interconnections, and functions in this early metazoan.

## Introduction

Life on earth has evolved from single cells to the stunning complexity and diversity that we see today. The transition from free-living single cells to multicellular organisms is a critical landmark in this progression (Nielsen, 2008). Choanoflagellates, protozoans with motile flagella, were one of the unicellular forms that adapted to multicellular way of life and eventually evolved into the first ancestor of the animal kingdom (King, 2004). Metazoa is thus a monophyletic group with a single transition from unicellular to multicellular giving rise to the entire diversity seen among animals (King, 2004; Nielsen, 2008). Within metazoa, tissue-grade organisation is first seen in phylum Cnidaria, which has true epithelia that keep the interior of the organism separate from the exterior (Nielsen, 2008). Appearance of a nervous system that enabled synchronised responses to stimuli, two germ layers, and a defined body axis and symmetry for the first time, are other features that make Cnidaria a milestone phylum in evolution (Boero et al., 2007; Bosch, 2008).

**Figure 1:**
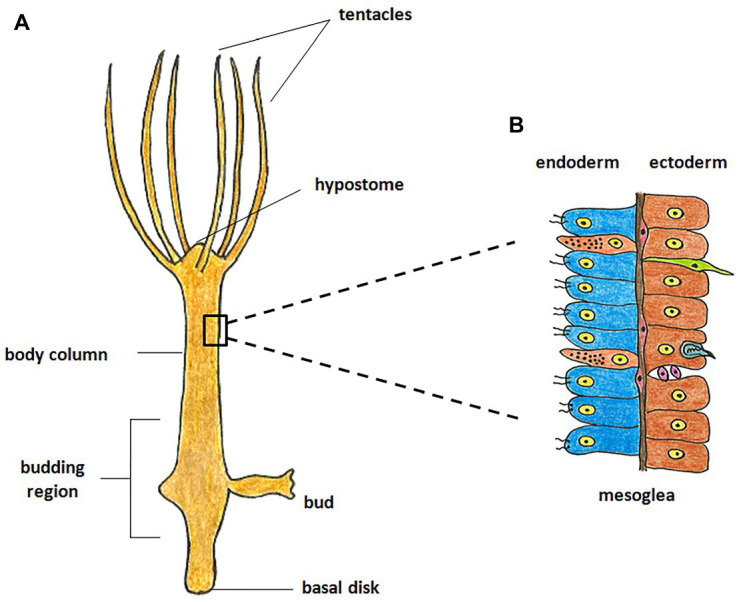
Morphology of hydra. **(A)** The polyp is cylindrical with an oral-aboral axis. The oral end consists of a conical hypostome surrounded by tentacles while the aboral end has a basal disk which enables it to attach to a substratum. **(B)** The body is composed of two germ layers, the inner endoderm and the outer ectoderm separated by mesoglea. The endoderm is composed of endodermal epithelial cells (blue) and gland cells (orange), while the ectoderm is composed of ectodermal epithelial cells (brown), interstitial stem cells (purple), neurons (green), and nematocytes (blue).

**Figure 2:**
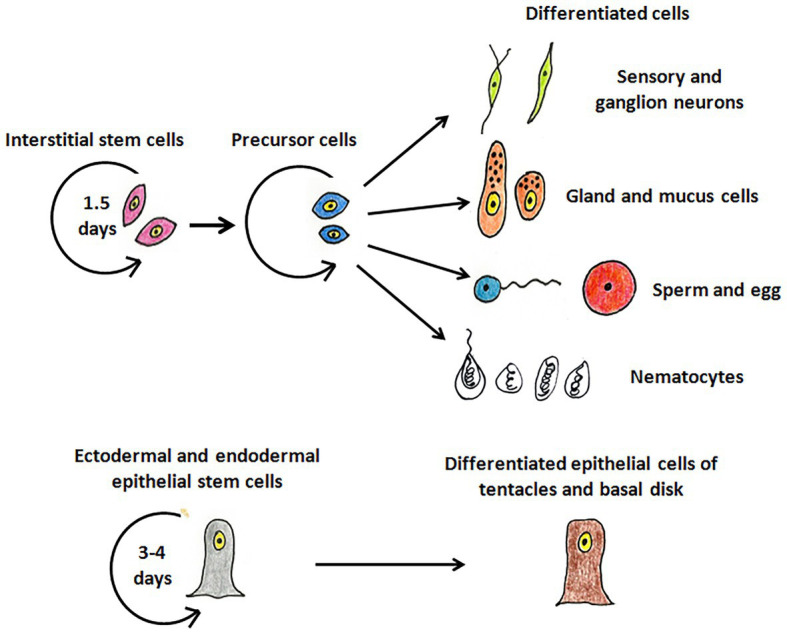
The stem cell lineages of hydra. The interstitial stem cells give rise to different types of differentiated cell types – nerve cells (green), mucus and gland cells (orange), gametes (blue and red), and nematocytes (white). The ectodermal and endodermal stem cells replenish cells of the ectoderm and endoderm, respectively, and give rise to terminally differentiated cells of tentacles and basal disk.

Hydra, first described by Abraham Trembley in 1744, is the most well-studied member of Cnidaria and has been used as a classic model system to investigate various phenomena. Hydra is a freshwater polyp found in stagnant or slow moving water bodies (Galliot and Schmid, 2002). It has a simple cylindrical body with radial symmetry and a defined oral-aboral axis. The oral end bears a conical hypostome surrounded by a whorl of tentacles and an opening at the apex, while the aboral end has a mucus-secreting basal disc that attaches to the substratum (Figure 1A). Hydra is diploblastic, consisting of a double-layered body wall of outer ectoderm and inner endoderm (Figure 1B) enclosing the gastrovascular cavity (Bode, 2003). It has around 30,000–200,000 cells of about 20 different types distributed across its two tissue layers. All cell types are derived from three stem cell lineages namely, ectodermal and endodermal epithelial stem cells (both unipotent), and the multipotent interstitial stem cells, which produce all other cell types (Figure 2; David, 1973; Bode, 1996). Hydra has a simple nerve net all over the body with a greater concentration of neurons in head and foot. While respiration, excretion and osmoregulation occur at the cellular level, digestion in hydra occurs both intracellularly and extracellularly in the body cavity (Bode, 1996). Well-fed hydra reproduces asexually by budding (Otto and Campbell, 1977) but sexual reproduction occurs in response to unfavourable conditions like starvation or decrease in temperature (Fujisawa, 2003).

## Hydra: an Intriguing Model Organism

Several salient features of hydra physiology make it a very useful model for teaching and research (Ghaskadbi, 2020a,b).

### Tissue Dynamics and Axial Patterning

Morphogenesis and pattern formation, which normally occur only in the embryonic stages, are active in hydra throughout its life (Müller, 1996). Cells continuously proliferate in the body column and move apically or basally without significant change in the animal’s size (Campbell, 1967a,b). To maintain tissue homeostasis, the excess cells generated are either sloughed off from the extremities or form a bud in a well-fed animal, effectively replacing all cells in the polyp within 20 days (Bode, 1996; Müller, 1996). Patterning processes are thus continuously active in hydra. Newly formed cells undergo position-specific changes in morphology and function as they move along the body column (Siebert et al., 2008). This has been ascribed to the presence of an organiser in the hypostome and morphogenetic gradients (Broun and Bode, 2002; Bode, 2009). Tissue dynamics and morphogenesis in hydra are thus tightly regulated, and the hydra polyp is, therefore, often called a ‘perpetual embryo’.

### Regeneration

Hydra has a tremendous capacity for regeneration. A polyp cut into two or more parts regenerates the missing regions in the same orientation as original. A piece of body column as small as 0.2 mm in diameter with a minimum of ~300 epithelial cells is able to regenerate the entire organism, as long as it contains both ectodermal and endodermal epithelial stem cells (Shimizu et al., 1993). Even groups of dissociated hydra cells are able to reaggregate and organise themselves to form a complete animal (Gierer et al., 1972). Regeneration in hydra is morphallactic, i.e., occurring by reorganisation of existing cells without new cell division, at least in the initial stages. The excised piece first reforms the missing parts and then grows by cell division to attain the normal size (Bode, 2003). The mechanisms that maintain normal pattern formation also regulate the formation of missing body parts during regeneration (Bode, 2009).

### Lack of Senescence

Hydra appears to show a complete lack of organismal senescence and negligible rates of natural mortality. Individual hydra and cohorts derived from them have been observed for over 4 years without any increase in mortality or decline in the rate of asexual (budding) or sexual reproduction. This is ascribed to its tissue dynamics, as individual cells are either continuously dividing or lost by sloughing or budding. Considering the rates of cell division and replacement, in 4 years, the epithelial cells of a single polyp would have divided ~300 times and all cells of the body would be completely replaced over 60 times, but still the cell division rates remain unchanged. Evolutionary theory of senescence posits that average life span of an organism is positively correlated to the age at beginning of reproduction (Martínez, 1998). Given that hydra begins budding in merely 4–5 days, the lack of senescence is remarkable. Some studies suggest that mortality increases in certain strains after sexual reproduction, but this may be regarded as an almost ‘irreversible depression’ brought on by intensive gamete production (Yoshida et al., 2006; Watanabe et al., 2009; Sun et al., 2020). No rise in mortality is observed in species like *Hydra vulgaris* AEP even after repeated rounds of sexual reproduction. Most evidence thus points to absence of ageing and potential immortality in hydra. However, the mechanisms by which the stem cells of hydra escape cellular senescence remain to be investigated.

### Unique Position in Evolution

The unique features of phylum Cnidaria (Boero et al., 2007) make hydra an invaluable model system in evolutionary studies. Genes involved in several developmental mechanisms and signalling pathways of hydra have orthologues in higher phyla (Bosch and Khalturin, 2002; Galliot and Schmid, 2002; Ghaskadbi, 2020a). Genes with triploblast-specific functions in higher organisms are present in hydra, hinting at possible origins of triploblasty. Moreover, Kortschak et al. (2003) have shown that many vertebrate genes, which are not represented in invertebrates like *Drosophila*, are present in Cnidarians, further highlighting the need to look beyond conventional model systems.

Availability of an EST database and sequence analysis platform (Hemmrich and Bosch, 2008) and sequencing of the *Hydra magnipapillata* genome (Chapman et al., 2010) provided further impetus to hydra research. Molecular techniques such as generation of transgenics (Wittlieb et al., 2006), RNA interference (Lohmann et al., 1999; Miljkovic-Licina et al., 2007), and microarrays (Hwang et al., 2007) are now being utilized to address fundamental questions in hydra biology. While several aspects like regeneration, pattern formation, signalling mechanisms, etc., are being intensively studied (Galliot, 2012; Ghaskadbi, 2020a), DNA repair in hydra is an area that has received scant attention.

## DNA Repair – an Overview

DNA is constantly subjected to attacks by various exogenous and endogenous agents causing different types of DNA damage, such as single- and double-strand breaks, formation of abasic sites or DNA adducts like cyclobutane pyrimidine dimers (CPDs) and 6-4 photoproducts, and generation of mis-paired bases (Hakem, 2008; Rastogi et al., 2010). Lesions in the DNA hamper cellular processes, and their accumulation over time may lead to cancer, trigger cellular senescence or programmed cell death (Hoeijmakers, 2001; Hakem, 2008). Cells have evolved several simple and complex DNA repair pathways to rectify injuries to DNA and restore integrity of the genome (Hakem, 2008).

**Figure 3:**
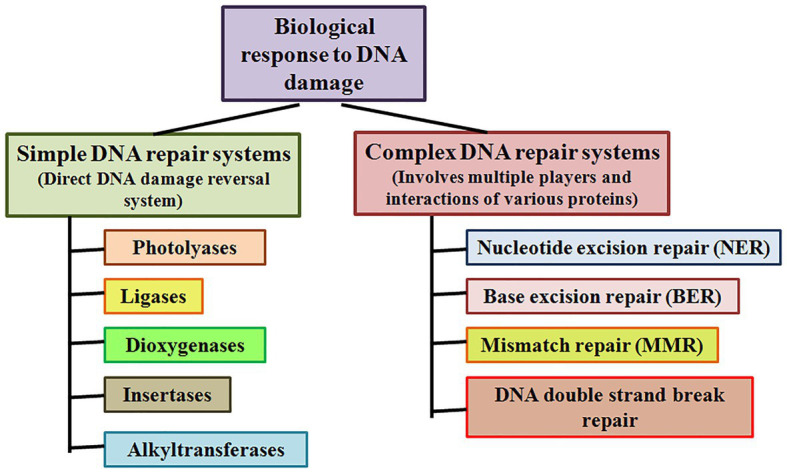
Simple and complex DNA repair systems. Simple DNA repair systems involve a single protein which is capable of direct reversal of DNA damage, while complex DNA repair systems involve multiple proteins leading to repair of the damaged DNA.

**Figure 4:**
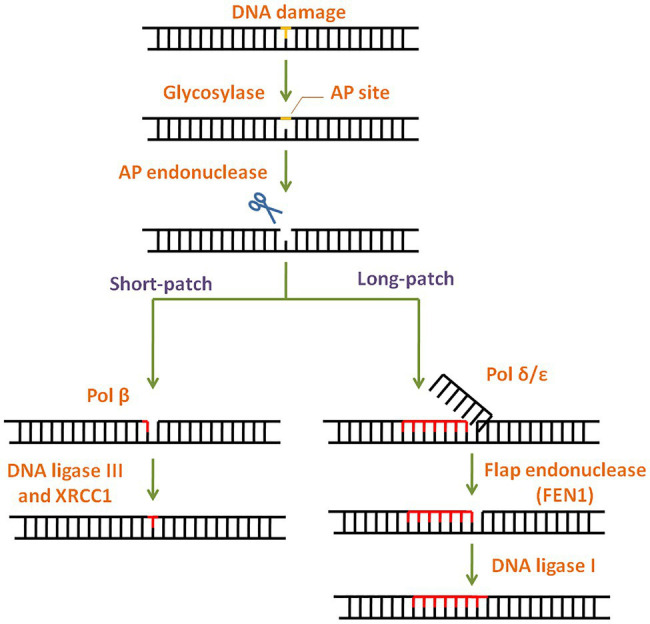
General scheme of the base excision repair (BER) pathway. In BER, the damaged base is recognised and removed by a base specific glycosylase generating an abasic site. Apurinic/apyrimidinic (AP) endonuclease then cleaves the phosphodiester backbone at the AP site. Depending on the number of damaged bases, short-patch or long-patch BER is initiated by DNA polymerase and completed by DNA ligase.

**Figure 5:**
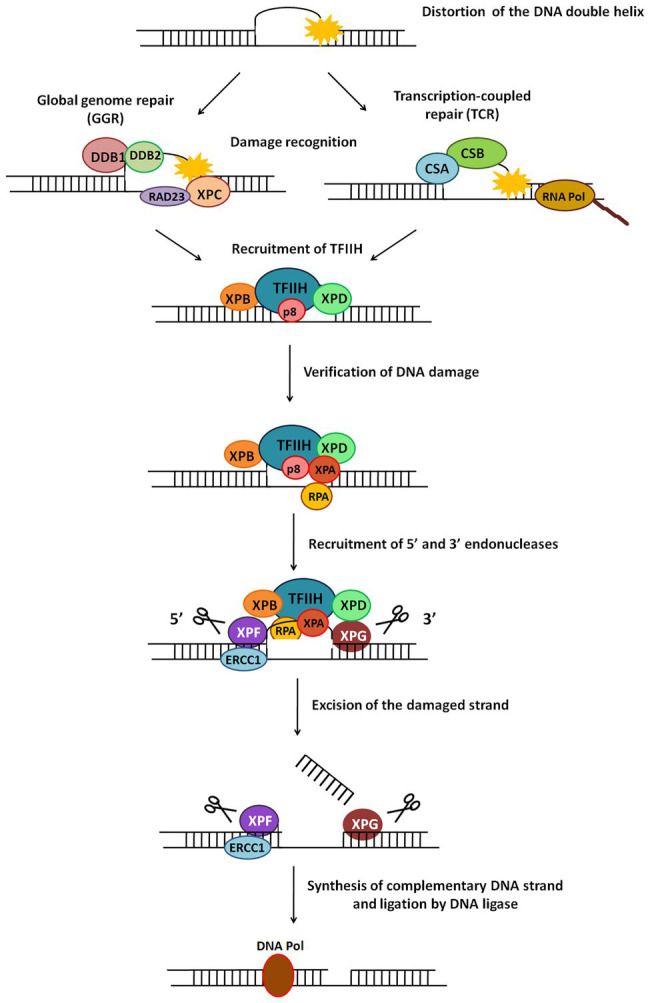
General scheme of the nucleotide excision repair (NER) pathway. NER repairs damage which causes distortion in DNA. There are two types of NER – global genome NER (GGR) or transcription-coupled NER (TCR). The major steps in this pathway include recognition of damage, recruitment of TFIIH, verification of DNA damage, recruitment of 5' and 3' endonucleases, excision of damaged strand followed by strand synthesis by DNA polymerase and ligation by DNA ligase.

Simple or single enzyme DNA repair systems (Figure 3) range from ***insertases*** that reinsert missing bases and ***ligases*** that seal nicks to ***alkyltransferases*** and ***dioxygenases*** that deal with large adducts and light-dependant ***photolyases*** that break bonds formed between adjacent nucleotides of the same strand (Jacobs et al., 1972; Livneh and Sperling, 1981; Eker et al., 2009; Rastogi et al., 2010). Complex DNA repair systems (Figure 3) are multi-enzyme pathways involving cutting and rejoining of the DNA backbone. ***Mismatch Repair (MMR)*** removes mis-incorporated bases during replication, specifically from the daughter strand (Modrich, 2006). ***Double Strand Break Repair*** operates either by homologous recombination where undamaged DNA is used as template, or by non-homologous end joining where uncapped free DNA ends are directly joined (Lieber, 2010). ***Excision Repair Pathways***, which work by ‘excising’, i.e., removing the damaged region from one strand and replacing it with a damage-free patch, are another category of complex repair pathways. They include base excision repair (BER; Figure 4) and nucleotide excision repair (NER; Figure 5), and will be the main focus of this review.

### Base Excision Repair

Base lesions generated by deamination, alkylation, depurination, and oxidation are repaired *via* BER with concerted action of different enzymes (Wilson and Kunkel, 2000). BER pathway is highly conserved throughout evolution (Robertson et al., 2009) and has two subtypes: short patch BER and long patch BER (Figure 4). The modified lesion is recognised and removed by a base specific DNA glycosylase, generating an apurinic/apyrimidinic (AP) site in DNA. This is recognised by AP endonuclease 1 (APE1), which cleaves the DNA backbone upstream of AP site. The damaged patch is removed and gap is filled by DNA polymerase and ligase. APE1 is the key regulator of BER pathway (Tell et al., 2009). Interestingly, mammalian APE1 has an additional redox activity, attributed to the cysteines in its N-terminal domain. This activity is involved in maintaining transcription factors in a reduced (active) state and regulation of cell proliferation.

### Nucleotide Excision Repair

This is a versatile mode of DNA repair that recognises and repairs helix-distorting or thermodynamically destabilizing lesions in DNA (de Boer and Hoeijmakers, 2000; Scharer, 2008). Since it detects changes in physical DNA structure, it is able to contend with an assortment of lesions like CPDs, 6-4 photoproducts, bulky adducts, etc., and repairs damage caused by UV radiation, mutagens, chemotherapeutic drugs, and more (Gillet and Scharer, 2006; Hakem, 2008). The NER pathway is a cut-and-patch mechanism (Figure 5) which removes a stretch of damage-containing region from one strand of DNA and replaces it using the other strand as a template. NER is present across all organisms from bacteria to humans, with similar overall nature of the pathway (damage recognition – incision – repair synthesis) but completely different sets of proteins in prokaryotes and eukaryotes (Hoeijmakers, 1991; Truglio et al., 2006). There are two sub-pathways of eukaryotic NER called global genome NER (GGR) and transcription coupled NER (TCR).

In humans, defects in NER proteins cause autosomal recessive disorders, such as xeroderma pigmentosum (XP), Cockayne syndrome (CS), trichothiodystrophy (TTD), etc. (Cleaver, 2000). XP is characterised by an extreme sensitivity to sunlight, dry parchment-like skin with abnormal pigmentation, very high susceptibility to skin cancers, and at times, ocular and neurological abnormalities (Cleaver, 2000; de Boer and Hoeijmakers, 2000). Most major proteins of the NER pathway are in fact named after the XP syndrome, as XP patients can be categorised into separate complementation groups (XPA-XPG and XPV), depending on the specific NER gene that is affected (Diderich et al., 2011).

## Biochemistry of NER

Broadly, NER involves the following major steps: damage recognition and verification, formation of open complex with recruitment of relevant proteins, incision on either side of the damage, and gap filling and ligation (Gillet and Scharer, 2006).

In prokaryotes like *Escherichia coli*, NER is carried out by the UvrA, B, C, and D proteins. UvrA_2_B complex recognises, binds to and unwinds the DNA around the damage. UvrC along with UvrB incises the DNA ~7 nucleotides upstream and 3–4 nucleotides downstream of the lesion. UvrD unwinds the DNA to release the damage-containing patch. The resulting gap is filled by DNA polymerase I and sealed by DNA ligase (Truglio et al., 2006).

In eukaryotic NER, a 24–32 nucleotide damage-containing stretch of DNA is removed and replaced (de Laat et al., 1999). The two sub-pathways of NER, GGR, and TCR, differ only in their mode of damage recognition and later converge to carry out subsequent steps by a common process (Figure 5; Hanawalt, 2002).

### Damage Recognition in GGR

In GGR, XPC protein in complex with human homolog of Rad23-B (hHR23B; Figure 5) scans the entire genome to identify helix-distorting lesions, especially CPDs, 6-4 photoproducts, cis-platin, and other chemical adducts. It then opens the DNA to facilitate the assembly of other larger proteins, including the transcription factor II-H (TFIIH) complex. In some cases, it is aided by the UV-damaged DNA binding (UV-DDB) complex which contains the *XPE* gene product (Figure 5). This recognition step culminates in recruitment of TFIIH to the lesion site (de Laat et al., 1999; Gillet and Scharer, 2006).

### Damage Recognition in TCR

RNA polymerase II (polII) stalls during transcription upon encountering adducts and distorting lesions, and itself acts as a recognition factor in TCR to trigger recruitment of NER proteins (Figure 5). TFIIH – a component of the polII complex – is a dual transcription and repair factor containing two DNA helicases (Figure 5; de Laat et al., 1999). CSA and CSB (Cockayne syndrome A/B) are also thought to be involved in binding and displacing RNA polII from the lesion (Figure 5; Fousteri and Mullenders, 2008).

### Verification of Damage and Open Complex Formation

XPB (3'→5' helicase) and XPD (5'→3' helicase) present in the TFIIH complex unwind an approximately 20–25 base pair region around the damaged site. This ATP-dependent catalytic step is probably an irreversible point at which both NER sub-pathways converge, and can be considered as damage verification (Figure 5; Gillet and Scharer, 2006). Replication protein A (RPA) then binds to the undamaged strand of the unwound DNA, while XPA, which has a high affinity for the damaged DNA strand, interacts with other proteins to form a fully opened, stabilised NER pre-incision complex of around 30 bases (Saijo et al., 1996; Cleaver and States, 1997).

### Recruitment of Nucleases and Dual Incision

Two endonucleases, XPF and XPG, cut the damaged DNA strand on either side of the damage (Figure 5). XPG joins pre-incision complex and structurally stabilises it (Gillet and Scharer, 2006). XPF, which forms a heterodimer with excision repair cross-complementing protein-1 (ERCC1), is recruited by XPA (Park and Sancar, 1994) and makes an incision at about 22–25 bases on 5' side of the lesion. XPG cuts at about 3–5 bases on 3' side of the damage, resulting in release of an excised damage-containing fragment of ~25–30 bases (Huang et al., 1992).

### Repair Synthesis and Ligation

5' Incision by XPF-ERCC1 results in 5'→3' overhang that is extended by polymerization (Staresincic et al., 2009). Replication factor C (RFC) and proliferating cell nuclear antigen (PCNA), both recruited by RPA, are required for repair synthesis along with either DNA pol δ or DNA pol ε. Polymerase fills the gap left by the excised patch, ligase seals the remaining nick (Figure 5), and the new patch of DNA rapidly associates with histones (Gillet and Scharer, 2006).

## Open Questions in NER Research

Though the molecular mechanisms of NER are well-worked out, diverse pathologies observed in XP patients and expression patterns of NER genes in various situations are not readily explained. While the connection between NER deficiency and cancer is justifiable, it is difficult to connect developmental and neurological abnormalities to defects in DNA repair proteins. Moreover, it is not clear why TCR deficiencies lead to neural abnormalities while those in GGR do not (Cleaver et al., 2009; McKinnon, 2009). Many NER proteins are shown to have additional roles in processes other than NER. XPF-ERCC1 is also involved in interstrand cross-link repair (Rahn et al., 2010), double strand break repair (Ahmad et al., 2008), and optimising the silencing of imprinted genes during development (Chatzinikolaou et al., 2017), while XPG is implicated in base excision repair (Klungland et al., 1999). Both XPF and XPG are involved in transcription, histone modification, and chromatin looping and remodelling (Le May et al., 2010a,b, 2012; Chatzinikolaou et al., 2017). CSB is additionally important for nucleosome remodelling and basal transcription (Fousteri and Mullenders, 2008; Cleaver et al., 2009). As part of TFIIH, XPB and XPD are involved in RNA polII-dependent transcription as well as repair (Hoogstraten et al., 2002). Also, many NER factors are recruited to active promoters even in absence of DNA damage, further indicating alternative roles (Le May et al., 2010a,b), though the detailed mechanism of their action remains to be elucidated.

## Evolution of Nucleotide Excision Repair

Genes and proteins implicated in NER have been reported across all five kingdoms of the living world. **Bacteria** have a functional NER pathway comprised of the UvrABC genes. The pathway follows broad steps analogous to eukaryotic NER, but the Uvr genes do not share significant homology with eukaryotic counterparts (Truglio et al., 2006). A large number of **Archaea** possess homologs of the UvrABC genes, but some also have homologs for eukaryotic genes such as XPB, XPD and XPF, whose functions remain to be fully elucidated (White, 2003; White and Allers, 2018). Among eukaryotes, the **Fungi** exemplified by yeast species *Saccharomyces cerevisae* and *Schizosaccharomyces pombe* have the full complement of NER genes that show similarity with human counterparts (Prakash and Prakash, 2000). NER genes have also been reported from the kingdom **Plantae**, characterised in the popular plant model *Arabidosis thaliana* (Kunz et al., 2005). The pathway is the most well-studied in kingdom **Animalia**, with NER genes characterised from several phyla. A fully operational NER is present in the nematode *Caenorhabditis elegans* and is in fact, differentially regulated in different tissues and at different life stages (Lans and Vermeulen, 2011). Among insects the pathway has been reported in *Dropsophila* (Sekelsky et al., 2000). Historically, most NER studies have taken place in mammals – from rodents to humans. Several NER-deficient cell lines from the Chinese hamster and mouse have been assigned to various excision repair cross-complementation (ERCC) groups as per their specific defects, and XP patient-derived cell lines have contributed to most of our understanding of human NER (de Boer and Hoeijmakers, 2000). Besides these reported studies, homology searches have indicated the presence of NER genes in several other animals, including echinoderms, amphibians, fish, birds, other mammals, etc.

### DNA Damage and Repair in Early Metazoans

Effect of known DNA damaging agents on Cnidarians such as corals has been reported. Coral bleaching is becoming increasingly prevalent due to rise in temperature and increased UV exposure in the coral reefs (Banaszak and Lesser, 2009). Formation of CPDs, UV-induced oxidative stress with expression of p53, and DNA breaks have been documented in various species of corals upon exposure to UV (Lesser and Farrell, 2004; Baruch et al., 2005; Banaszak, 2007). Planula larvae of the coral *Porites astreoides* were unable to survive 48 h of exposure to benzo(a)pyrene [B(a)P; Farina et al., 2008], while microsomes from sea anemone *Bunodosoma cavernata* produced oxidative metabolites upon incubation with the chemical (Winston et al., 1998). These studies suggest that a functional repair pathway is necessary in these organisms, especially since UV and B(a)P induced DNA damage is known to be repaired by NER. However, the NER pathway remains largely uncharacterised in early-evolved animal phyla including Cnidaria, with only a few sporadic reports of NER genes from early metazoans. For instance, *XPB* from the marine demosponge *Geodia cydonium* is light-inducible, with its expression increasing a massive 29 times as compared to control, upon exposure to UVB (Batel et al., 1998).

In this context, it is interesting to study DNA repair in a basal metazoan like hydra. Considering the unique position of Cnidaria as a sister group to bilaterians, the study of repair pathways in hydra can provide clues to evolution of DNA repair systems in multicellular animals as well as roles of individual proteins in various processes. Given hydra’s well-studied developmental processes and simple nervous system, it may be a useful model to study potential developmental roles of NER proteins and aetiology of neural abnormalities in NER related disorders. The apparent lack of ageing and longevity of its stem cells, makes analysis of repair pathways in hydra even more interesting. Thus, studying DNA repair in Cnidarians is relevant for understanding both – physiology of early metazoans and evolution of DNA repair systems. We analyse here the nature of NER and BER pathway genes in hydra in context of their functions and their relationships to homologs from other organisms.

## UV-Induced DNA Damage and Repair in Hydra

**Figure 6:**
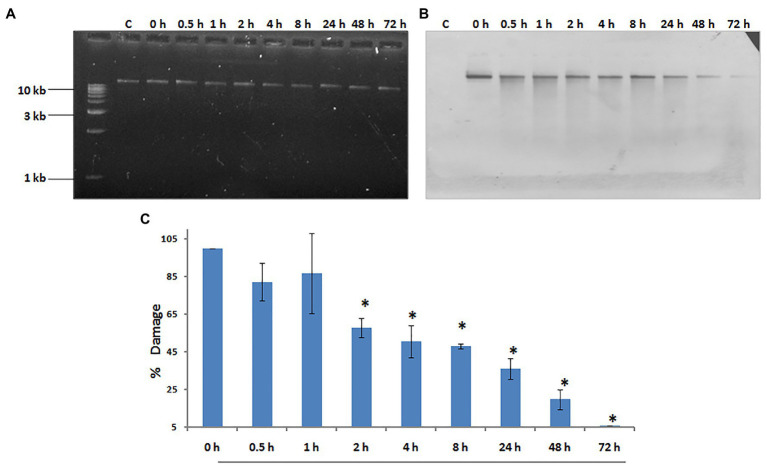
Hydra repairs cyclobutane pyrimidine dimers (CPDs) induced by UV. About 1% agarose gel (input) observed under UV transilluminator **(A)** with 250 ng each of genomic DNA samples prepared after UV exposure at 500 J/m2, followed by recovery periods of 0, 0.5, 1, 2, 4, 8, 24, 48, and 72 h, respectively. **(B)** DNA blot assay detected by nitro blue tetrazolium (NBT) and 5-bromo-4-chloro-3-indolyl-phosphate (BCIP) to determine repair of CPDs in hydra post-UV radiation at different recovery times of 0, 0.5, 1, 2, 4, 8, 24, 48, and 72 h, respectively. **(C)** Quantitation of removal of CPDs by hydra. Percent DNA damage and recovery time post-UV radiation were plotted on *y*-axis and *x*-axis, respectively. Each experiment was repeated three times and data presented are ±SE of these replicates. ^*^p < 0.05 as compared to control. Reproduced from Galande et al. (2018), with permission.

Our laboratory has shown that exposure to 500 J/m^2^ of UV radiation induces budding in whole hydra, and causes duplication of the foot region in regenerating middle pieces of trisected hydra (Ghaskadbi et al., 2005; Krishnapati et al., 2016). This implied that exposure to UV can affect pattern formation in hydra, and the same dose of UV was used to examine DNA repair. Genomic DNA of UV-exposed hydra was extracted at sequential recovery time points between 0 and 72 h post-exposure, and checked for repair of CPDs using a monoclonal anti-CPD antibody (Figure 6A). Most of the CPDs were repaired by 72 h, confirming that hydra possesses the capacity to repair damaged DNA (Figures 6B,C). This observation indicates that DNA repair capacity arose early in metazoans, and may also have implications in the context of widespread UV-induced coral bleaching that is affecting the fragile coral reef ecosystems globally (Galande et al., 2018).

## NER Pathway Genes in Hydra

### XPA: Damaged DNA Binding and Open Complex Formation

XPA, the rate limiting factor of NER, is a small monomeric protein without which repair does not take place (Aboussekhra et al., 1995; Cleaver and States, 1997; Kang et al., 2010a). XPA-RPA complex ensures that repair occurs only at the appropriate site (Thoma and Vasquez, 2003). Apart from dermatological symptoms, some XP-A patients show ocular, neurological, and cognitive dysfunctions, which are not explained solely on the basis of its role in NER (Anttinen et al., 2008).

XPA has several functional domains – zinc-finger to bind to DNA and domains for interaction with RPA, TFIIH, and XPF-ERCC1 complex (reviewed in Cleaver and States, 1997). *XPA* gene seems to be present only in eukaryotes (White, 2003) and shows no homology to UvrA, the bacterial damage recognition protein (Hoeijmakers, 1991). Our group first reported the presence of *XPA* gene in hydra as a 774 bp predicted CDS that was amplified and cloned from *H. vulgaris* Ind-Pune strain (GenBank accession no. JN411719; Barve et al., 2013a).

#### Sequence Analysis of Hydra XPA Suggests Conservation of Function

Hydra XPA contains the conserved nuclear localization signal, which marks proteins to be sent to the nucleus. It shows two well-conserved Pfam domains – XPA-N and XPA-C – with six critical cysteine residues in the all-important DNA-binding zinc-finger motif conserved between hydra and human XPA. Hydra XPA also has the RPA-binding domain, with high degree of conservation in the 24 amino acid region required for binding to the RPA-70 subunit. The interaction between XPA and N-terminal region of ERCC1 is essential for NER, and occurs through two regions of XPA: the well-conserved and critical G motif and the more varied E motif (Li et al., 1995). Hydra XPA contains three of the four amino acids in the human XPA G motif, but hydra E motif is variable (Barve et al., 2013a). Two cysteines in the C-terminal region of XPA that are thought to be involved in interaction with TFIIH (Park et al., 1995; Cleaver and States, 1997) are conserved in hydra XPA. Hydra XPA thus appears to have all the necessary domains for interaction with its various partners. The predicted structure of a part of hydra XPA protein has a very high similarity with the solved structures of corresponding region of human XPA, with a very good fit upon superimposition and a low root mean square deviation (RMSD) value. This suggests that based on presence of functional domains and structure, hydra XPA probably functions similar to its human and other homologs (Barve et al., 2013a).

Putative hydra XPA protein sequence showed notable similarity with XPA from various animals, with up to 52% identity and 70% similarity at the amino acid level. Notably, the predicted hydra XPA protein shows higher similarity with XPA from many mammals and other vertebrates like fish, frog, chick, rather than invertebrates such as the fruit fly. Phylogenetic trees constructed for XPA protein showed a similar pattern. The cluster of hydra and echinoderm XPA consistently grouped with the chordate XPA cluster, and invertebrates including *Drosophila* and *C. elegans* were outside this branch (Barve et al., 2013a). The *XPA* gene has not yet been reported from the Archaea or in the single-celled eukaryote, *Plasmodium*, indicating that it may have arisen in more complex eukaryotes (White, 2003). The observation that hydra XPA clusters with deuterostome sequences is in line with previous findings from hydra and *Nematostella*, that Cnidarian genes often show high similarity with vertebrate counterparts (Kortschak et al., 2003; Chapman et al., 2010; Reddy et al., 2011; Barve et al., 2013b). XPA thus appears to be conserved across species at not just sequence but also at structure level, thus reiterating its early evolution and critical function (Barve et al., 2013a).

### XPB, XPD: The 5' and 3' Helicases

XPB and XPD, the two helicases that are part of the TFIIH complex, unwind the DNA around the lesion in NER. Mutations in these genes affect several cellular processes such as transcription, NER, and chromosome segregation (Compe and Egly, 2016). Indicating its indispensability, only a few XPB mutations have been identified till date, probably because they cause embryonic lethality (van Brabant et al., 2000). Our lab has identified and isolated *XPB* (GenBank accession no. JN411718.2) and *XPD* (GenBank accession no. JN411716.2) homologs from *H. vulgaris* Ind-Pune for the first time (Galande et al., 2018).

#### Hydra XPB and XPD Contain all Relevant Domains and Are Functional Helicases

**Figure 7:**
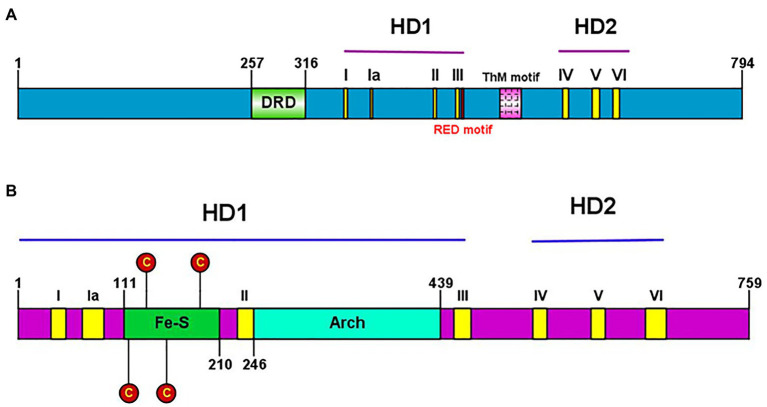
**(A)** Presence of conserved helicase domains and motifs in hydra XPB (HyXPB). Pictorial depiction of the domain structure of HyXPB using IBS Illustrator. HD1 and HD2 are the helicase domains and the classical seven helicase motifs (yellow) are numbered as I, Ia, II, III, IV, V, and VI. DRD (green) stands for the damage recognition domain, ThM (pink) is the thumblike motif, and RED motif (red) is made of the arg-glu-asp conserved residues. **(B)** Presence of conserved helicase domains and motifs in hydra XPD (HyXPD). Pictorial depiction of the domain structure of HyXPD using IBS Illustrator. HD1 and HD2 are the helicase domains and the classical seven helicase motifs (yellow) are numbered as I, Ia, II, III, IV, V, and VI. Fe-S domain (green) contains the four essential cysteine residues (red) and the arch domain (turquoise blue) is involved in CDK-activating kinase (CAK) assembly. Reproduced from Galande et al. (2018), with permission.

Conserved helicase motifs found in various proteins are classified into six superfamilies, SF1–SF6 (Fairman-Williams et al., 2010). Hydra XPB and XPD, each contain two helicase domains (HD1 and HD2), and have seven helicase motifs of the SF2 type spanning these two domains (Figure 7). Hydra XPB also possesses two domains that are conserved from archaea to humans – a damage recognition domain (DRD) important for its NER function, and a thumb-like (ThM) domain (Figure 7A) containing conserved basic amino acids such as arginine and lysine (Galande et al., 2018). The RED motif (Fan et al., 2006) essential for DNA unwinding is also present in hydra XPB (Figure 7A). In addition to the HD1 and 2 domains, hydra XPD has two other auxiliary domains – Fe-S domain and arch domain – within HD1 (Figure 7B). Similar to XPDs from other organisms (Liu et al., 2008), hydra XPD Fe-S domain contains four key conserved cysteine residues while the arch domain contains basic amino acids arginine and lysine which are essential for its interactions with the other components of TFIIH (Galande et al., 2018; Peissert et al., 2020; Figure 7B). Hydra XPB and XPD thus seem to have all the domains essential for their function and share close similarities with respective homologs from other animals.

**Figure 8:**
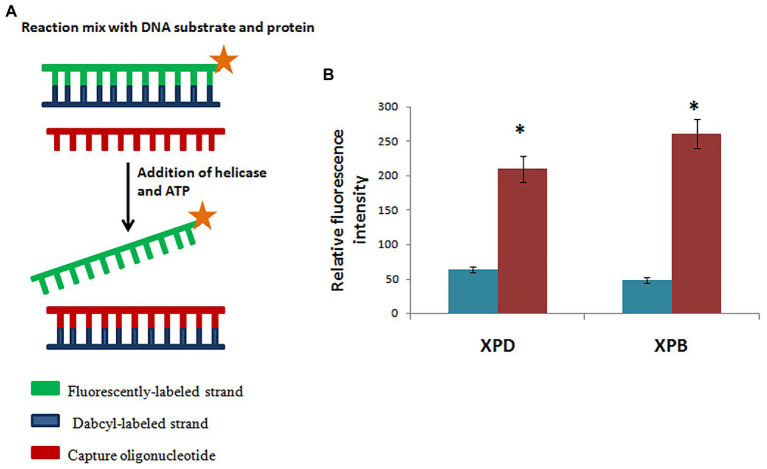
Hydra XPB and HyXPD are able to unwind DNA. **(A)** Schematic representation of helicase assay. Since HyXPB and HyXPD unwind DNA in opposite polarities, cy3 was labelled at one end and dabcyl as a quencher on the opposite strand. Capture oligonucleotide was added to bind to dabcyl-labelled strand to prevent it from annealing to cy3 labelled strand. **(B)** In comparison to control (without HyXPB), a 5-fold increase in fluorescence is observed, whereas HyXPD shows a 3-fold increase in fluorescence as compared to its control. Reproduced from Galande et al. (2018) with permission. *p < 0.05 as compared to control.

Homology modeling of hydra XPB and XPD based on human XPB and XPD templates from the solved structure of TFIIH complex (Greber et al., 2017; PDB 5OF4) revealed close similarity of hydra proteins to their human counterparts. Phylogenetic analysis showed close relation of hydra XPB and XPD with *Drosophila* and vertebrate homologs, indicating their early evolution (Galande et al., 2018). Assay for helicase activity using fluorescently-labelled substrate demonstrated that recombinant hydra XPB unwinds substrate in the 3'→5' direction, while recombinant hydra XPD unwinds in the 5'→3' direction (Figure 8A). This confirms polarity-dependent helicase activity for both proteins (Figure 8B), similar to homologs from other animals. mRNA expression analysis by quantitative RT-PCR and whole-mount *in situ* hybridization showed that expression of *hydra XPB* and *XPD* remained unaltered post-UV exposure, indicating constitutive expression of the genes (Galande et al., 2018).

#### Hydra XPB and XPD Fail to Complement Respective Deficient Human Cell Lines as They Do Not Get Incorporated Into a Functional TFIIH Complex

Considering the close similarity of hydra XPB and XPD with their human counterparts, ability of the hydra homologs to complement the defect in human XPB and XPD deficient cell lines was examined. XPB deficient human cell lines transfected with hydra XPB showed only 4.5% survival upon exposure to UV, as against 78% survival in controls transfected with human XPB. Similarly, the post-UV exposure survival rate was just 0.87% in XPD-deficient human cells transfected with hydra XPD in comparison with 76% survival in human XPD transfected controls (Galande et al., 2018). Thus, hydra XPB and XPD failed to complement defects in human cells. XPB and XPD function as subunits of the TFIIH complex (Compe and Egly, 2016). TFIIH is composed of two subcomplexes – CORE (consisting of p62, p44, p34, p52, p8, and XPB) and CDK-activating kinase (CAK; made up of MAT1, Cyclin H, and CDK7) – bridged by XPD. p62 and MAT1 from the CORE and CAK complex, respectively, are known to interact with XPB and XPD in TFIIH (Compe and Egly, 2016). After they failed to complement XP-deficient human cell lines, co-immunoprecipitation studies were conducted with hydra XPB and XPD and human p62 and MAT1 to find out if a fully functional TFIIH was being assembled in the first place. Hydra XPB co-immunoprecipitated with p62 but not with MAT1, whereas hydra XPD did not co-immunoprecipitate with either. These results indicate that hydra XPB and XPD do not get incorporated into a functional TFIIH in XPB and XPD deficient human cells, explaining why complementation may not have worked. This points towards differences in key amino acid residues which prevented important protein-protein interactions essential for the functionality of TFIIH complex (Galande et al., 2018).

### XPF: Incision on 5' Side of the Lesion

The interaction of XPF with ERCC1 is crucial for its stability, and its recruitment and positioning at the NER site. The heterodimer cuts the damaged DNA strand on 5' side of the lesion during NER (Sijbers et al., 1996), and is demonstrated to have several other roles as well. Mutations in the *XPF* gene can also lead to a segmental progeroid syndrome of spontaneous accelerated ageing (Niedernhofer et al., 2006). Our group described the *XPF* gene in hydra for the first time. We cloned a 2,451 bp stretch of *XPF*, containing 5' UTR and sequence encoding 816 amino acids, from *H. vulgaris* Ind-Pune (GenBank accession no. HQ380893; Barve et al., 2013b).

#### Hydra XPF Contains all Essential Functional Domains and Is Very Similar to Its Vertebrate Counterparts

About 69.8% of hydra *XPF* nucleotide sequence is (A+T), which is in keeping with the (A+T)-rich nature of hydra genome (Chapman et al., 2010). Two nuclear localisation signals (NLS) are present in hydra XPF, indicating that the protein can be imported to its site of action, i.e., the nucleus. Hydra XPF has a conserved nuclease motif-containing ERCC4 domain, making it a part of the evolutionarily ancient XPF/MUS81 protein family whose members are found from Archaea and earliest eukaryotes (Ciccia et al., 2008). Hydra XPF’s ERCC4 domain contains a ‘GD XnV/IERKX3D’ motif, the defining feature essential for its endonuclease activity (Enzlin and Scharer, 2002). Two helix-hairpin-helix (HhH)_2_ domains present at C-terminal regions of both XPF and ERCC1 are responsible for their dimerization. In human XPF, seven amino acids from the (HhH)_2_ domain form a hydrophobic pocket which interacts with ERCC1 (Tripsianes et al., 2005; Ciccia et al., 2008). Three of these seven residues are conserved between hydra and human XPF, while three more are substituted conservatively, suggesting that hydra XPF may also form a hydrophobic pocket at its C-terminus. Additionally, ~10 conserved acidic residues important for binding to the metal cation required for XPF’s catalytic action (Enzlin and Scharer, 2002) are also present in hydra XPF. These *in silico* analyses indicate that hydra XPF can function as an endonuclease in NER.

Two solved crystal structures covering two critical regions of XPF (ERCC1-binding domain and ERCC4 domain) are available: chain B of human XPF-ERCC1 complex (Tripsianes et al., 2005; PDB id: 1Z00), and *Pyrococcus furiosus* XPF endonuclease domain (Nishino et al., 2003; PDB id: 1J23). Superimposition of computationally predicted hydra XPF structures with the solved ones showed a very good fit and very low RMSD values, further suggesting that it is a functional protein. Moreover, computational predictions of human and *Drosophila* XPF are also similar to the solved structures, indicating early evolution and conservation of XPF across phyla and even across kingdoms (Barve et al., 2013b).

#### Hydra XPF Clusters With Early Metazoan and Vertebrate Counterparts in Phylogenetic Analysis

Predicted amino acid sequence of hydra XPF closely matched with homologs from various animals. Similar to other hydra proteins, hydra XPF sequence matched better with vertebrate rather than invertebrate homologs, corroborating previous reports on similar lines (Kortschak et al., 2003; Chapman et al., 2010). In phylogenetic trees, the cluster of early metazoan XPFs (two hydra species, another Cnidarian, and a placozoan) consistently grouped with the deuterostome XPF cluster (echinoderm, hemichordate, and vertebrate). *Drosophila*, *C. elegans*, and *Trypanosoma* lay outside this early metazoan-deuterostome XPF cluster. Moreover, the extended branch lengths for these protostome XPFs indicated that they have diverged substantially from ancestral metazoan sequences, possibly due to rapid genome change in some of these organisms (Barve et al., 2013b).

#### Predominant Expression of XPF in Interstitial Stem Cells May Have Implications for Germ Line Protection

**Figure 9:**
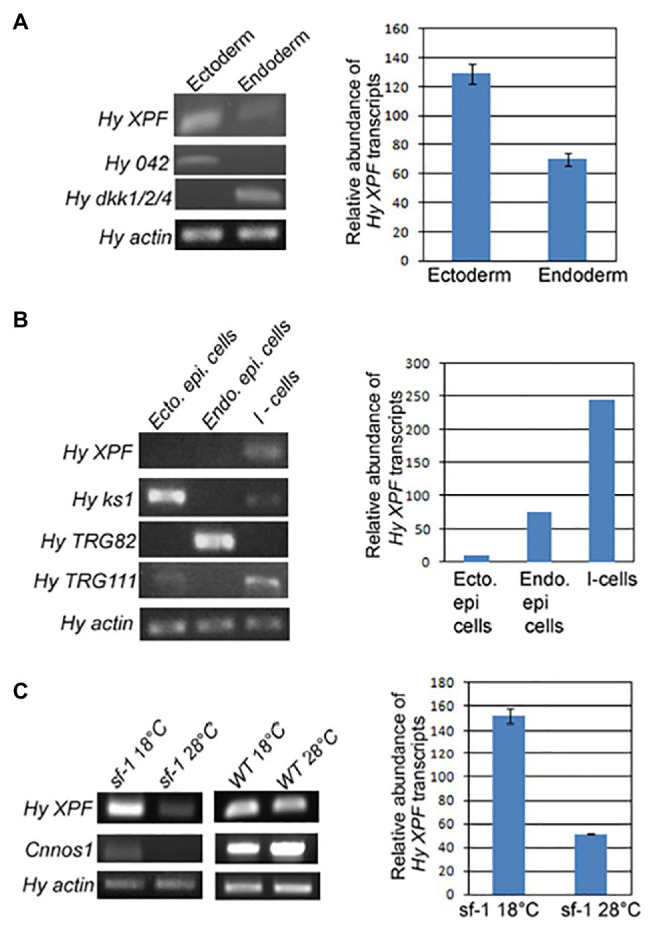
Analysis of expression pattern of hydra XPF by semi-quantitative RT-PCR. Hydra actin was used as housekeeping control for equilibration in each set. Histogram depicting relative abundance of XPF transcripts among the samples is shown adjoining each RT-PCR set. **(A)** Level of XPF mRNA is nearly twice in ectoderm as compared to endoderm. Ectodermal marker Hy042 and endodermal marker HyDkk1/2/4 are expressed predominantly in the respective samples, indicating that the two tissue layers are cleanly separated. **(B)** XPF expression was compared among three cell types of hydra and was found to be predominant in interstitial cells. Levels of ks1, TRG82, and TRG111, markers for ectodermal epithelial cells, endodermal epithelial cells, and interstitial cells, respectively, indicate the purity of each cell type fraction. **(C)** Prominent expression of XPF in interstitial cells was confirmed using sf-1 hydra. XPF expression reduced by nearly 3-fold upon exposure of sf-1 to 28°C, but remained unchanged in wild-type (WT) hydra. Levels of interstitial stem cell marker Cnnos1 dropped drastically in sf-1 hydra kept at 28°C indicating loss of interstitial cells but were unaffected in WT hydra, demonstrating that a decrease in XPF levels is directly correlated with loss of interstitial cells. Reproduced from Barve et al. (2013b).

Expression of *XPF* analysed by whole mount *in situ* hybridization was strongest in the central region of the hydra body column. Semi-quantitative RT-PCR showed that *XPF* is expressed substantially more in the ectoderm of hydra than in the endoderm (Figure 9A). When expression of *XPF* was compared across three main stem cell types of hydra – ectodermal epithelial, endodermal epithelial, and interstitial cells – it was found to be predominant in the interstitial cells (Figure 9B). The results were confirmed using sf-1, a temperature sensitive variant of *H. magnipapillata*, that loses its interstitial cells at non-permissive temperature (Terada et al., 1988). In interstitial cell-depleted hydra, expression of *XPF* as seen by semi-quantitative RT-PCR, decreased almost 3-fold, showing direct correlation between the number of interstitial cells and the level of *XPF* expression (Figure 9C; Barve et al., 2013b). This observation also aligns with an earlier finding that hydra interstitial cells express high levels of *FoxO* (Bridge et al., 2010). Members of FoxO family are implicated in protective and stress-response pathways, such as promotion of longevity (Greer et al., 2007), protection from oxidative stress (Kops et al., 2002), and response to cellular stress by inducing DNA repair (Tran et al., 2002).

Since interstitial stem cells reside in the ectoderm, this may also explain the higher *XPF* levels observed in the outer layer. Interstitial stem cells produce many cell types including gametes in hydra, and with their derivatives they make up ~80% of all cells in the polyp. Of the three stem cell types, interstitial cells cycle at the fastest rate (Bode, 1996). Hence, high expression of repair genes like *XPF* could ensure that a well-preserved genome is maintained in the tissues of the potentially immortal polyp and passed on to the buds. Unlike several other animals, germ cells are not segregated in hydra, as interstitial cells constitute the germ line (Bode, 1996). Thus, high expression of *XPF* – and by extension NER genes – in these cells could be a means to protect the germline genome (Barve et al., 2013b).

### Other NER Genes in Hydra

Our group has identified, cloned, and sequenced cDNAs of several other NER genes from hydra: *XPC*, *XPE*, *XPG*, *CSA*, *CSB*, and *TTDA*. Coding sequences (partial or full-length) of these genes have been submitted to the NCBI GenBank database (Table 1). Taken together, these observations strongly suggest that the NER pathway is functional in hydra, suggesting that it arose early in evolution and has been highly conserved.

**Table 1:** Accession numbers of CDS of other NER genes identified from hydra.

Gene	Role in DNA repair	GenBank accession number
*Hydra XPC*	Damage recognition, open complex formation in GG-NER	JN411717
*Hydra XPE*	Damage recognition in GG-NER	JN41715
*Hydra XPG*	3' Endonuclease of NER	KC503882
*Hydra CSA*	Damage recognition in TC-NER (?)	JQ822227
*Hydra CSB*	Damage recognition and recruitment of NER factors in TC-NER	JQ822228
*Hydra TTDA*	Essential for stability of TFIIH complex	MG593807.1

#### NER Genes in Placozoa, Porifera, and Cnidarians Other Than Hydra

Protein sequences of XPA, XPB, XPD, and XPF homologs from early metazoans such as a placozoan, a demosponge, three corals, and two anemones were used to analyse their similarity with hydra homologs (Table 2). We observed close identity and similarity of these proteins with their counterparts from hydra indicating early origins of these essential repair proteins and of the NER pathway.

**Table 2:** Similarities of hydra xeroderma pigmentosum (XP) proteins with those from other basal metazoans.

XP protein	Name of the organism	Number of amino acids	Percent identity with Hydra Protein	Percent similarity with Hydra Protein
XPA Binds to damaged DNA and verifies the lesion	*Trichoplax adhaerens* (Placozoan)	266	44.3	77.0
	*Amphimedon queenslandica* (Sponge)	281	38.2	72.1
	*Stylophora pistillata* (Coral)	267	51.7	81.3
	*Pocillopora damicornis* (Coral)	272	44.0	71.0
	*Nematostella vectensis* (Sea anemone)	266	53.0	79.3
	*Acropora millepora* (Coral)	247	48.2	82.0
XPB Core component of TFIIH, 3'→5' ATP-dependent helicase	*Trichoplax* sp. H2	799	68.0	88.8
	*Amphimedon queenslandica*	664	64.7	85.9
	*Stylophora pistillata*	777	69.9	87.2
	*Pocillopora damicornis*	792	71.2	88.9
	*Nematostella vectensis*	796	70.5	88.4
	*Acropora millepora*	794	71.0	89.7
XPD Bridges the core and the CAK complexes of TFIIH, 5'→3' ATP-dependent helicase.	*Trichoplax sp.* H2	761	69.4	91.1
	*Amphimedon queenslandica*	754	63.6	88.5
	*Stylophora pistillata*	759	76.6	91.9
	*Pocillopora damicornis*	759	76.9	92.6
	*Nematostella vectensis*	735	75.7	92.8
	*Acropora millepora*	759	77.7	91.8
	*Exaiptasia diaphana* (Sea anemone)	782	74.1	88.6
XPF DNA endonuclease which incises damaged strand at 5' junction of the NER bubble.	*Trichoplax sp.* H2	846	45.1	75.6
	*Amphimedon queenslandica*	917	40.7	70.9
	*Stylophora pistillata*	917	46.8	73.0
	*Exaiptasia diaphana*	849	43.4	69.6
	*Nematostella vectensis*	915	47.5	73.8
	*Acropora millepora*	918	48.3	73.0

## BER Pathway Genes in Hydra

Base excision repair removes modified bases from DNA which could otherwise induce mutations by mispairing. APE1 is a critical component of the BER pathway. Mammalian APE1 plays a dual role due to the presence of two functional domains – a C-terminal DNA repair domain and an N-terminal redox domain. The redox function of APE1 is ascribed to the presence of three conserved cysteine residues in the N-terminal domain. Interestingly, the redox function is not observed in APE1 from other animals such as *Drosophila*, zebrafish, frog, etc., which lack the three crucial cysteines in their N-terminal domain (Georgiadis et al., 2008).

**Figure 10:**
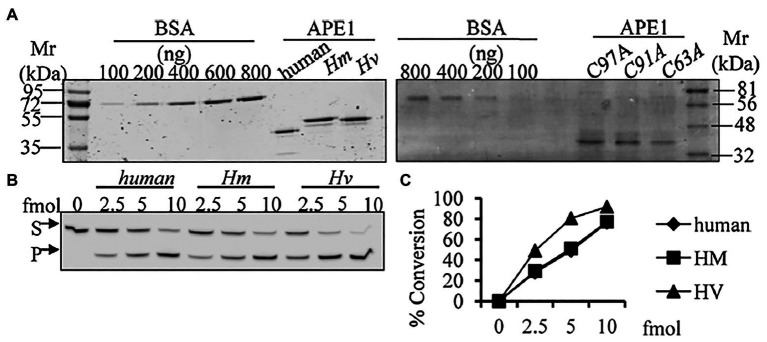
Purified HyAPE1 shows AP endonuclease activity. **(A)** Separation of HyAPE1, human recombinant APE1, and mutants C93A, C91A, and C97A HvAPE1. **(B,C)** AP-site incision activity of human and HyAPE1 using AP-endonuclease activity assay. Data in **(C)** are average values ± SD of three independent experiments. Reproduced from Pekhale et al. (2017), with permission.

**Figure 11:**
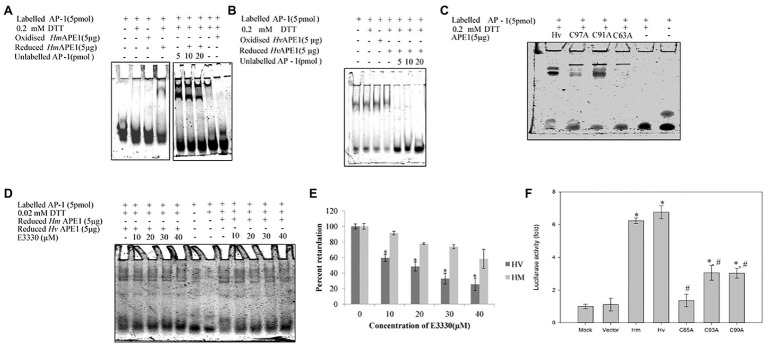
Purified HyAPE1 shows redox activity. **(A,B)** Redox activity of HyAPE1. **(C)** Redox activity of HvAPE1 mutants C63A, C91A, and C97A. **(D)** Inhibition of redox activity of APE1 by specific redox inhibitor E3330. Data in **(E)** are average values ± SD of three independent experiments for redox activity of HyAPE1 in presence of E3330 (^*^p < 0.05 compared to control HyAPE1). **(F)** Effect of HyAPE1 and its cysteine mutants C63A, C91A, and C97A on NFκB transactivation (^*^p < 0.05 compared to control; ^#^*p* < 0.05 compared to HvAPE1). Each experiment was repeated at least three times and data presented are average ± SE of all these replicates. Reproduced from Pekhale et al. (2017), with permission.

We have identified, cloned, and characterised the hydra homolog of APE1, as a 975 bp cDNA coding for 324 amino acids (Pekhale et al., 2017). The endonuclease activity of this protein was demonstrated by an assay for cleavage of an AP site in labelled dsDNA that produced a 14 bp incised product (Figure 10). Remarkably, *in silico* analysis showed that the three cysteine residues critical for redox function are conserved in hydra APE1. Redox activity of hydra APE1 was confirmed by its ability to reduce the transcription factors AP-1 and p53, which then bind to their DNA substrate and retard it in an electrophoretic mobility shift assay (Figure 11). To further confirm the role of the three cysteines in redox activity, three recombinant hydra APE1 proteins were produced, each with one of the three main cysteines mutated. Mutation of C63 led to loss of hydra APE1’s redox activity while mutation of the other two cysteines (C91 and C97) reduced it to an extent (Figure 11). This redox activity is specifically inhibited by E3330, which also inhibits regeneration in hydra polyps, hinting at a connection between redox activity of APE1 and regeneration. Further supporting this connection, exposure to excess hydrogen peroxide simultaneously inhibited regeneration and APE1 expression, along with accumulation of DNA damage (Haval et al., 2020).

Thus, the APE1 homolog in hydra is fully functional with both endonuclease as well as redox activities. Akin to the findings for NER pathway genes, hydra APE1 also seems to be more similar to its vertebrate (here mammalian) counterpart. Moreover, the results demonstrate that the redox activity of APE1 is evolutionarily ancient, and not a recent acquisition as previously believed (Georgiadis et al., 2008; Pekhale et al., 2017).

## Summary and Conclusion

### DNA Repair in Hydra – Relevance for Evolution of Repair Proteins and Pathways

**Figure 12:**
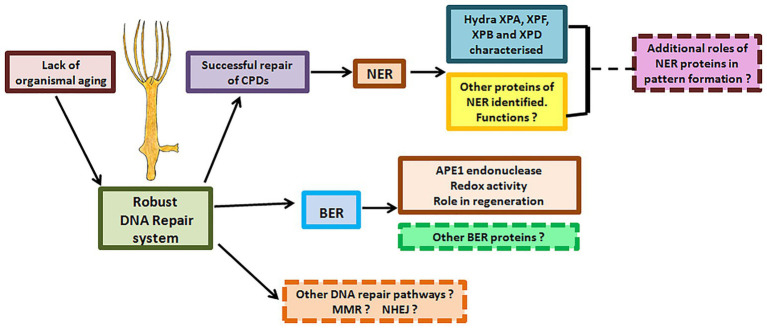
DNA repair proteins and their possible roles in hydra. Hydra possesses robust DNA repair mechanisms that perhaps allow its stem cells to pass on an error-free genome to subsequent generations, explaining its lack of senescence and immortality. Apart from the DNA repair proteins characterised so far, other DNA repair proteins and pathways in hydra remain to be identified. Possible roles of DNA repair proteins in regeneration and pattern formation also remain to be studied.

The critical nature of the versatile NER pathway is underlined by the fact that it is conserved from bacteria to man. Though functionally analogous, NER pathways in prokaryotes and eukaryotes differ significantly with hardly any sequence similarity between their respective NER proteins (Hoeijmakers, 1991). In a significant contrast, the archaea have elements similar to both eukaryotic and prokaryotic DNA repair pathways (White and Allers, 2018), indicating that the eukaryotic NER pathway may have ancient archaeal origins. Rudiments of eukaryotic NER were thus probably present even before the last eukaryotic common ancestor (LECA). Within eukaryotes, data from multiple genes indicate similarity of DNA repair proteins at sequence, domain, structure, and possibly functional levels, showing that the pathways are highly conserved. However, there are also some differences. For example, NER of fungi and animals differs in the way repair proteins are assembled at the site (Volker et al., 2001; Hoogstraten et al., 2002; Guzder et al., 2006). Among animals, the presence of classical NER and BER pathways has now been established in hydra, demonstrating that they arose early in their present form in this kingdom. While there are divergences such as absence of TCR in *Drosophila* cell lines (de Cock et al., 1992), the presence of *CSA* and *CSB* in hydra underlines the evolutionary antiquity of this NER sub-pathway. The surprising observation that hydra APE1 possesses redox activity, which is absent in most animals other than mammals, again points to early evolution of features of repair proteins that were thought to be new acquisitions. Additionally, the interesting connection between the BER gene *APE1* and regeneration warrants investigation and may lead to a better understanding of this fascinating process. Analysis of BER and NER in hydra, a member of an early-evolved animal phylum with true tissues and a nervous system, can thus provide a window into the evolution of these crucial pathways (Figure 12).

Remarkably, despite their great evolutionary distance, putative proteins of hydra NER genes appear to be more similar to vertebrate sequences than model invertebrates. Phylogenetic trees for individual NER proteins reiterated that Cnidarian homologs are more similar to deuterostome ones than *Drosophila* or *Caenorhabditis*. This suggests that genomes of these popular model organisms may have diverged substantially from ancestral sequences due to rapid evolution, while deuterostomes may have retained features of the basal metazoan lineage. While the analyses are constrained by lack of information about NER genes from other early metazoans and invertebrates, the data indicate the high complexity of Cnidarian genomes and their deep connections with other bilaterian lineages (Kortschak et al., 2003; Chapman et al., 2010). In view of the high divergence in genomes of common model invertebrates, the observed similarity of hydra genes to vertebrate homologs further highlights its significance as a model in the study of metazoan evolution and physiology.

### Hydra – A Good Model to Study Correlation Between DNA Damage, Cancer, and Ageing

Accumulation of DNA damage is linked with various age-associated diseases such as cancers, cardiovascular disorders, dementia, etc., and certain markers of genomic instability appear with increasing age (Sedelnikova et al., 2004; Petr et al., 2020). At the same time, expression of several DNA repair genes is higher in long-lived organisms such as humans and naked mole rats as compared to shorter-lived mice (MacRae et al., 2015). It is thus not surprising that, as seen in XP and CS patients, defective NER can cause both cancer (a disease typified by mutations and genetic instability) and premature ageing (Cleaver, 2000; Marteijn et al., 2014). As shown by presence of repair pathway genes as well as evidence of naturally-occurring transplantable tumors in hydra (Domazet-Lošo et al., 2014), both DNA repair and tumorigenesis have ancient evolutionary roots. The insights from this potentially immortal organism can, therefore, prove invaluable in understanding DNA damage and repair, and its implications for cancer and/or senescence.

The higher expression levels of *XPF* in the multipotent, gamete-producing interstitial stem cells could be indicative of more efficient DNA repair for rigorous maintenance of genome integrity in this important stem cell type. Organismal ageing is linked to ageing of somatic stem cells – an individual’s age can be directly correlated to the age of her/his stem cells (Ho et al., 2005). At the same time, accumulation of DNA damage triggers senescence at the cellular level, and leads to progressive deterioration of overall physiology and ageing (d’Adda di Fagagna, 2008; Hakem, 2008; Freitas and de Magalhães, 2011). The number of times a cell divides (de Magalhaes and Faragher, 2008) and the build-up of replication errors, both influence ageing. Stem cells of the ‘immortal’ hydra undergo numerous cell divisions but the animal does not age (Martínez, 1998), and the stem cells seem to avoid senescence. In absence of a clear mechanism to explain this escape from stem cell and organismal ageing, it is interesting to speculate on the significance of high expression of a DNA repair gene such as *XPF* in hydra’s interstitial stem cells.

## Future Perspectives

The findings so far advocate hydra as an excellent model to analyse metazoan-specific features of DNA repair, including developmental and neurological abnormalities seen in some patients and evolution of the alternative roles of repair proteins (Figure 12). For instance, NER proteins are thought to be involved in DNA demethylation, histone modification, and telomere maintenance (Barreto et al., 2007; Wu et al., 2008; Le May et al., 2010a). Examination of these phenomena in hydra may reveal interesting details of the epigenetic status of its cells, which cycle continuously without senescing or losing their stemness. Such a study could also provide insights on whether these other roles are the primary functions or secondary acquisitions of the respective repair genes.

Kang et al. (2010b) have shown that expression of *XPA* in humans varies in a circadian manner, and they ask whether this phenomenon is evolutionarily ancient. Hydra is photo responsive (Plachetzki et al., 2012), and another Cnidarian *Nematostella* has the complete circadian clock (Reitzel et al., 2010). Analysis of *XPA* expression in hydra could yield interesting information about evolution of circadian regulation of NER pathway among animals. Similarly, probing the role of *XPF* and other genes, in context of its high expression in interstitial cells, could yield important clues about the physiology of this interesting cell type. Analysis of spatio-temporal expression of individual NER and BER genes in hydra can thus provide important insights.

Much of what we know today about ageing is from model organisms such as yeast, fruit fly, mouse, and the worm *C. elegans*. Hydra can be a great model to study ageing and its relation with DNA repair, and exhaustion/senescence of stem cells. Hydra is thought to evade ageing by continuously replenishing its tissues through its three stem cell populations (Bode, 1996). But given the DNA damage theory of ageing, it is intriguing how the stem cells of hydra are able to continuously divide without apparently accumulating any errors. Investigating how DNA repair pathways work in its cells may provide clues about the mechanisms by which hydra stem cells escape senescence. With identification of the major players of NER and BER in hydra and the observed high expression of *XPF* in interstitial cells, the stage is now set for future studies in this direction.

## Author Contributions

SSG and SG conceived the idea of this article and approved and communicated the manuscript. AB, AG, SSG, and SG planned the outline and finalized the manuscript. AB and AG prepared parts of the first draft. AG prepared the original figures and tables. AB, SSG, and SG worked further on the draft. AB checked the draft for appropriate style. All authors contributed to the article and approved the submitted version.

### Conflict of Interest

The authors declare that the research was conducted in the absence of any commercial or financial relationships that could be construed as a potential conflict of interest.
